# Family-Based Benchmarking of Copy Number Variation Detection Software

**DOI:** 10.1371/journal.pone.0133465

**Published:** 2015-07-21

**Authors:** Marcel Elie Nutsua, Annegret Fischer, Almut Nebel, Sylvia Hofmann, Stefan Schreiber, Michael Krawczak, Michael Nothnagel

**Affiliations:** 1 Institute of Clinical Molecular Biology, Christian-Albrechts University, Kiel, Germany; 2 Institute of Medical Informatics and Statistics, Christian-Albrechts University, Kiel, Germany; 3 Cologne Center for Genomics, University of Cologne, Cologne, Germany; Emory University School Of Medicine, UNITED STATES

## Abstract

The analysis of structural variants, in particular of copy-number variations (CNVs), has proven valuable in unraveling the genetic basis of human diseases. Hence, a large number of algorithms have been developed for the detection of CNVs in SNP array signal intensity data. Using the European and African HapMap trio data, we undertook a comparative evaluation of six commonly used CNV detection software tools, namely Affymetrix Power Tools (APT), QuantiSNP, PennCNV, GLAD, R-gada and VEGA, and assessed their level of pair-wise prediction concordance. The tool-specific CNV prediction accuracy was assessed in silico by way of intra-familial validation. Software tools differed greatly in terms of the number and length of the CNVs predicted as well as the number of markers included in a CNV. All software tools predicted substantially more deletions than duplications. Intra-familial validation revealed consistently low levels of prediction accuracy as measured by the proportion of validated CNVs (34-60%). Moreover, up to 20% of apparent family-based validations were found to be due to chance alone. Software using Hidden Markov models (HMM) showed a trend to predict fewer CNVs than segmentation-based algorithms albeit with greater validity. PennCNV yielded the highest prediction accuracy (60.9%). Finally, the pairwise concordance of CNV prediction was found to vary widely with the software tools involved. We recommend HMM-based software, in particular PennCNV, rather than segmentation-based algorithms when validity is the primary concern of CNV detection. QuantiSNP may be used as an additional tool to detect sets of CNVs not detectable by the other tools. Our study also reemphasizes the need for laboratory-based validation, such as qPCR, of CNVs predicted in silico.

## Introduction

The term ‘copy number variation’ (CNV) refers to the recurrence of moderately sized stretches of DNA (>1 kb) that exhibit inter-individual differences in the number of times they occur in a genome [1,2]. Scientific interest in human CNVs has been stirred partly by the fact that only a minor proportion of the heritability of common complex diseases is explained by disease-associated single-nucleotide polymorphisms (SNPs) [3–5]. Other forms of genetic variation, including CNVs, are therefore likely to play an important role in the etiology of these diseases [3]. Correspondingly, CNVs have been implicated in various common disorders, including Crohn disease [6], rheumatoid arthritis and diabetes [7], psoriasis [8], intellectual disability [9], obesity [10], myocardial infarction [11], schizophrenia [12] and autism [13]. While CNV detection in the past was based solely upon aCGH or SNP array signal intensity data [14], technological progress of DNA sequencing today allows direct detection of CNVs [15], for example, in exomes [16]. However, genome-wide SNP array data still form an important basis of CNV detection, not the least due to their ample availability from past genome-wide association studies (GWAS). A re-assessment of phenotypic associations of CNVs in these large legacy sample collections is warranted and to be expected for the coming years.

A variety of software tools have been developed for the detection of CNVs in SNP array data. Depending upon the underlying mathematical model, these tools can be divided broadly into two classes, namely those implementing a Hidden Markov model (HMM) and those using a segmentation algorithm. In a nutshell, HMM-based approaches aim at predicting covert copy number (CN) states along a Markov chain whereas segmentation algorithms split chromosomes into segments and try to sensibly assign a CN state to each segment. The interpretation of the derived CN states also differs between algorithms because ‘state’ either refers to a nominal class or a numerical genotype. Thus, a copy number class merely indicates the type of variation, i.e. whether there is a gain or loss of genetic material, whereas a copy number genotype specifies the number of copies present in a diploid genome. All available HMM algorithms predict up to six different copy number genotypes whilst all segmentation algorithms predict copy number class as one of three different types.

Different approaches have been taken in the past to benchmark CNV detection software [17–21]. Using early Affymetrix 100K SNP data, Baross et al. (2007) [17] noted substantial false-positive prediction rates with software tools CNAG (Copy Number Analyzer for GeneChip) [22], dChip (DNA-Chip Analyzer) [23] and GLAD [24]. The same authors also reported a high variability of these tools in terms of the number of CNVs predicted. Winchester et al. (2009) [18] assessed the accuracy of CNV prediction for five other software tools, using data from the more recent Affymetrix Genome-Wide Human SNP Array 6.0 and Illumina 1M-Duo BeadChip chips. They compared their SNP-based results to those of previously published sequencing studies [1,25,26], but only in single HapMap samples. In any case, the Winchester et al. study revealed that a large number of predicted CNVs could not be confirmed by any previous publication (up to 80%, depending upon the software used), and that predictions differed greatly both between software tools and between confirmation studies. In the same vein, Zhang et al. (2011) [19] applied Birdsuite [27], Partek (Partek Inc, St. Loius, MO), HelixTree (Golden Helix, Inc) and PennCNV [28] to three different data sets and observed a positive correlation between the number of markers included in a CNV and the ‘recovery rate’, defined by the authors as the proportion of previously published, validated CNVs that were also detected in their own study. Interestingly, the recovery rate was found to be negatively correlated with CNV population frequency. The same study also revealed a low consistency of the CNVs predicted in eight samples previously analyzed by Kidd et al. (2008) [25] and Conrad et al. (2010) [2]. More recently, Eckel-Passow et al. (2011) [20] reported substantial variability of the pairwise concordance of CNV predictions by PennCNV [28], Affymetrix Power Tools (APT) [29], Aroma. Affymetrix [30] and CRLMM (Corrected Robust Linear Model with Maximum Likelihood Distance) [31]. An in-depth assessment of PennCNV and CRLMM revealed a median concordance of 52% for deletions and of 48% for duplications. More deletions than duplications were predicted by both tools, and the empirical false-positive prediction rates were as high as 26% for CRLMM and 24% for PennCNV. Pinto et al. (2011) [21] analyzed six samples on 11 different microarrays and predicted CNVs using as many different software tools including PennCNV and QuantiSNP. The data generated by each microarray platform was analyzed with one to five of these tools. The experiments were performed in triplicate for each sample, and the authors observed inter-software concordance of < 50% and a reproducibility in replicate experiments of < 70%.

None of the above studies used family data for CNV validation but instead relied upon experimental validation of a very limited set of CNVs, DNA sequencing information, or a concordant prediction made by different algorithms. Moreover, none of the studies paid any attention to population differences in CNV prediction, despite previous reports that such differences do exist [1,32–34]. A general conclusion has been that more than one software tool should be used synergistically to increase specificity, and that CNVs should be validated experimentally by more reliable methods such as qPCR. However, although many of the currently available software tools were included in at least one of the studies, no systematic comparison has yet been undertaken of the main characteristics of CNVs predicted by a given algorithm, including the length, marker density and inter-marker distance.

We therefore assessed in detail the performance of six commonly used software tools for CNV detection in Affymetrix SNP array data. The tools of interest included HMM-based algorithms APT [29], QuantiSNP [35] and PennCNV [28] in addition to segmentation-based algorithms R- gada [36], GLAD [24] and VEGA [37]. The SNP genotyping of APT is based on the birdseed algorithm of the well-known Birdsuite software package and can be seen as a extension of the Birdsuite approach. The Birdsuite software package was therefore not included in our comparison. We used publicly available SNP array signal intensity data from the International HapMap project [38–42] for CNV detection and a trio design for validation. Our results may guide future choices of CNV software for particular applications and should also instruct the interpretation of the results obtained.

## Materials and Methods

### Proband Data

We used signal intensity data of 60 trios (180 individuals) from the Affymetrix Sample Data Set, which were part of HapMap Phases 1 and 2 public releases 21a (released on 1st November 2007). Half of the trios were African (Yoruba in Ibadan, Nigeria, YRI) whereas the other half was of European ancestry (Utah Residents with Northern and Western European Ancestry, CEU). All samples had been genotyped with Affymetrix Genome-Wide Human SNP Array 6.0, which contains probes for 906,600 SNPs and an additional 945,826 CNV probes [43]. NetAffx annotation files (release 31, UCSC hg19) were used to map the markers on the chip to the human genome. The average genotyping call rate in the complete public release 21a data set (270 samples) was 99.83% (technical documentation) and the concordance with HapMap genotypes (release 21a) was 99.84%. Since there are no general CNV-specific quality control measures, we used all samples and applied software-specific default quality control if available (see below). We used the 60 unrelated offspring samples for CNV detection and the 120 parental samples for subsequent validation. Our analysis was confined to autosomes.

### CNV definition

In contrast to previous publications [1,2], we defined as a CNV any stretch of DNA that either has additional copies (duplication, gain) or is lacking (deletion, loss) compared to a reference genome, not restricting the CNV predictions by their size. We used the NetAffx Annotation File (release 31), containing marker positions according to the UCSC genome assembly version hg19 to annotate the predicted CNVs. The two copy number (CN) classes of ‘gain’ and ‘loss’ were subdivided into CN states according to the number of chromosomes (1 or 2) affected by the respective gain or loss.

### SNP array data

Depending upon technology, SNP arrays contain multiple probes for each of the two alleles (A and B) of a SNP. All probes specific to one allele are collectively called a ‘channel’. The intensity of each of the two channel signals (denoted R^A^ and R^B^) reflects the amount of genetic material hybridized. The signal ratio allows inference, not only of the SNP genotype, but also of the relative amount of genomic material present at the target locus. Affymetrix Human SNP Array 6.0 contains six to eight probes per SNP, corresponding to three to four probes per channel. The array also contains a large number of probes for regions that may contain CNVs, but not SNPs, and these probes directly measure the total amount of genetic material present [44,45].

Inference of the copy number status at a given locus is made by comparing the sum of the observed channel signals, *R* ^*obs*^ = *R* ^*A*^ + *R* ^*B*^, to its expectation *R* ^exp^. The definition of *R* ^*exp*^ varies between CNV detection algorithms. However, all algorithms use the marker-specific Log-2 Raw Data Ratio *LLR* = log_2_ *R* ^*obs*^-log_2_ *R* ^*exp*^ as the basic input to infer a CN state, although some also rely upon the B allele fraction (BAF). If a CNV is present, the BAF is notably different from 0 (genotype AA), 0.5 (AB) and 1 (BB). The BAF is derived from a transformation, *θ*_*i*_, of the sample-specific channel intensity ratios for the i^th^ sample, calculated as $2/\pi \cdot \text{arctan}(R_{i}^{A}/R_{i}^{B})$.

For each marker, this yields three genotype-specific median θ values, taken over all samples, namely *θ* ^*AA*^, *θ* ^*AB*^ and *θ* ^*BB*^. The BAF of an observed θ value is then calculated as

$$BAF=\{\begin{array}{l} 0,\text{ if }\theta <\theta^{AA}\\ 0.5\cdot (\theta -\theta^{AA})/(\theta^{AB}-\theta^{AA}),\text{ if }\theta^{AA}<\theta <\theta^{AB}\\ 0.5+0.5\cdot (\theta -\theta^{AB})/(\theta^{BB}-\theta^{AB}),\text{ if }\theta^{AB}<\theta <\theta^{BB}\\ 1,\text{ if }\theta \text{>}\theta^{BB} \end{array}$$

### Software tools

We studied six commonly used software tools for CNV detection in Affymetrix SNP array data, namely APT [29], QuantiSNP [35] and PennCNV [28], implementing an HMM algorithm, and segmentation-based tools R-gada [36], GLAD [24], and VEGA [37]. All programs were run with their default options unless stated otherwise, which also includes default quality control measures by the respective software (see S1 File for the used commands). CNVs were defined separately for each sample, ignoring familial relationships.

#### APT

The Affymetrix Power Tools (APT) [29] equate *R* ^exp^ to the median sum of the sample-specific-channel signals, taken over all markers, or use a pre-computed reference [46] to obtain marker-specific LRR values. A Hidden Markov model is then fitted to the sequence of LRR values along the genome to assign hidden copy number states. We used program apt-copynumber-workflow of the APT bundle (version 1.14.2) with default settings in the single-sample mode and option—text-output set to true. The pre-computed Copy Number Analysis HapMap Reference File (Release 31) was used as a reference and the NetAffx Annotation File (Release 31) was used for alignment (publically available at the Affymetrix website http://www.affymetrix.com/support/index.affx).

#### PennCNV

CNV analysis of Affymetrix data with PennCNV [28] follows the Penn-Affy protocol (http://www.openbioinformatics.org/penncnv/) according to which LRR and BAF are inferred from canonical genotype clusters [47] by means of linear interpolation. The genotype clusters are generated from genotype calls that have been obtained with the APT software (see above). The sequences of LRR and BAF values are used in an HMM algorithm to infer the hidden copy number states. The PennCNV 2011Jun16 version was included in our study. First, apt-probeset-genotype and apt-probeset-summarize of APT (version 1.14.2) were used for genotype calling and allele-specific signal extraction, as laid down in the protocol. Second, canonical genotype clusters [47] were generated using generate_affy_geno_cluster.pl, which is part of the PennCNV-Affy tool. Clusters were then used to calculate LRR and BAF values via linear interpolation with normalize_affy_geno_cluster.pl. The sequence of LRR and BAF values was analyzed using detect_cnv.pl with default parameters.

#### QuantiSNP

QuantiSNP [35] relies on pre-computed LRR and BAF values (e.g. from PennCNV) that are subjected to its own HMM algorithm to infer hidden copy number states. QuantiSNP (version 2) was applied according to the instructions given on the QuantiSNP project webpage (https://sites.google.com/site/quantisnp/). Signal files created with PennCNV were used as input.

#### R-gada

The segmentation algorithm implemented in R-gada [36] uses LRR values pre-computed along the genome and tries to find discontinuities by sparse Bayesian learning. For the resulting segments, the average LRR of all markers falling into the segment is compared to the median LRR of the respective chromosome. Based upon the outcome, a CN class is assigned to the segment. R-gada (version 0.8–5) was run using LRR values calculated with APT program apt-copynumber-workflow.

#### GLAD

The GLAD software [24] was developed for the analysis of aCGH data. However, since the program uses signal intensities for segmentation, it can also be applied to SNP array data. GLAD uses pre-computed LRR values and applies the Adaptive Weights Smoothing algorithm to find discontinuities along the genome. CN classes are then assigned depending upon the difference between the segment-specific median LRR and the median LRR closest to zero. GLAD (version 2.20.0) was run using R 2.15 and APT-derived LRR values (see above).

#### VEGA

The segmentation algorithm implemented in the VEGA software [37] is based upon the Mumford and Shah model [37] and uses pre-computed LRR values as well. After segmentation, CN states are assigned to the resulting segments depending upon whether mean LRR is smaller or larger than zero. VEGA (version 1.7.0) was run using R 2.15 and APT-derived LRR values (see above).

### Standardization of output

While PennCNV, QuantiSNP, R-gada and VEGA report a list of segments and their respective CN state, APT and GLAD output a list of markers and their CN states. To allow comparison between tools, we converted all output to lists of segments, if not provided by the software itself. We also summarized CN genotypes into CN classes with three possible states per segment (‘normal’, ‘gain’ or ‘loss’). Since some algorithms (e.g., PennCNV) do not support sex-chromosomal analyses by default, we considered only autosomal CNVs. All autosomal CNV predictions including outliers < 1kb were retained for the benchmark.

### CNV benchmarking

We evaluated the six software tools in terms of both the characteristics of the predicted CNVs (i.e. their number, length and type) and the validity of the predictions made. We also compared the marker density within those CNVs that were detected in the 60 unrelated children from trios. The presence or absence of a CNV in the parents was used to validate each prediction in the offspring, since the overwhelming majority (up to 99%) of all CNVs in a genome is inherited [44,48] and will also be present in one of the parental genomes. While de-novo CNVs do exist, they play only a minor role. More specifically, a CNV detected in an offspring was considered validated if one or more segments of the same CN class (i.e. ‘gain’ or ‘loss’) that covered >90% of the offspring CNV were found in at least one parent. We also applied other thresholds for the required overlap. All analyses were repeated separately in the African (YRI) and European (CEU) samples to recognize possible population differences in terms of CNV detection. To assess the likelihood of a CNV being validated by chance alone following our family approach, we randomly reassigned parents to offspring and repeated this procedure ten times. This analysis was carried out twice, once drawing parents from a joint pool of CEU and YRI trios and once considering CEU and YRI trios separately.

The possible influence on CNV prediction of the underlying mathematical model was assessed by comparing the median of the outcome variables of interest for the three HMM-based programs (APT, PennCNV, QuantiSNP) to those for the three segmentation-based programs (GLAD, R-gada, VEGA). Again, to evaluate possible population differences, we repeated our analyses separately for the CEU and YRI samples. Finally, we investigated the inter-software concordance in terms of CNV prediction by considering the proportion of CNVs predicted by one tool that were also found by the other tool, using only validated CNV predictions in this approach. A CNV predicted by one tool was considered verified by the other if >90% of the CNV was assigned the same CN state by the second tool. We also considered other thresholds for the necessary overlap. Additionally, we generated a sample-specific set of CNVs concordantly called by at least three algorithms and compared each tool to this call set.

External verification data based on other technologies than SNP genotyping were obtained from the Database of Genomic Variants (DGV, http://dgv.tcag.ca, build 37, release 2013-07-23). We used a sequencing-based set of variants containing the results of 12 studies [26,49–58] (“DGV sequencing”) and employed a 90% verification threshold.

All statistical analyses were performed with R 2.15.2. Outcome differences between software tools were tested for statistical significance using a pairwise Wilcoxon signed-rank test.

## Results

### CNV prediction

**Fig 1:**
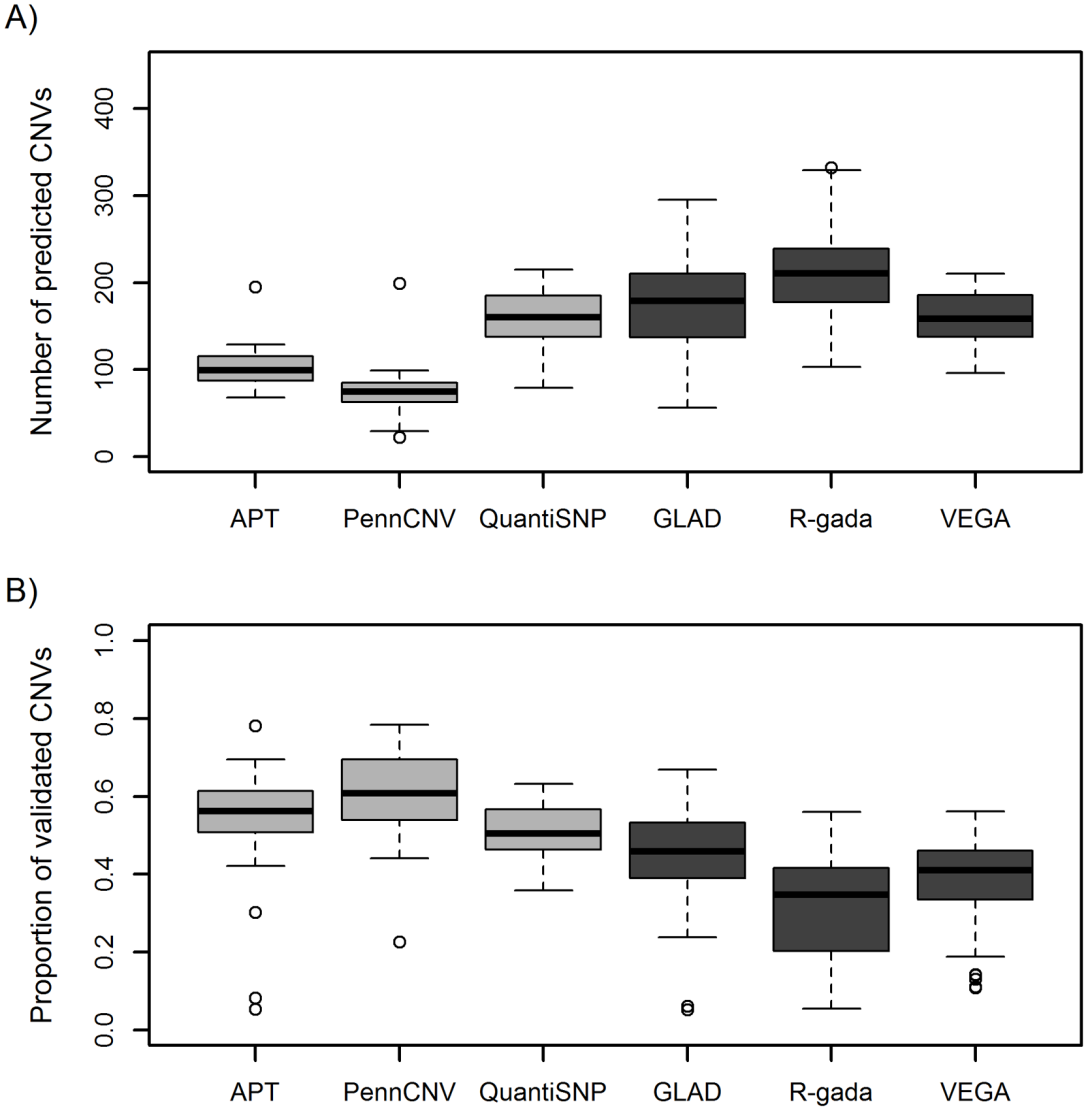
CNV prediction and family-based validation. **A:** Number of CNVs predicted per sample. **B:** Proportion of CNVs per sample validated by parental information. **Light grey:** HMM-based algorithms; **dark grey:** segmentation algorithms.

The spectra of CNVs predicted by different programs varied widely, both in terms of their number and length and of the marker density within CNVs. In the offspring of the 60 European (CEU) and African (YRI) HapMap trios, the median CNV number ranged from 75 per sample, predicted by PennCNV, to 211 per sample for R-gada (Fig 1 and Table 1). Segmentation algorithms predicted significantly more CNVs than HMM algorithms (median: 182 vs. 98, Wilcoxon signed rank test p = 1.6×10–11) and showed a (non-significant) trend towards a higher inter-software variability in CNV number (median absolute deviation 42.3 vs. 19.3, p = 0.12 from 10,000 permutations of class labels). All software except PennCNV predicted fewer CNVs in Europeans (CEU) than in Africans (YRI, p<0.05 for all tools; S1 Table).

**Table 1:** Sample-specific features of predicted CNVs.

Software	Total number	Median length [kb]	Median cumulated length [Mb]	Median number of Markers in CNV	Median inter-marker distance [kb]	DDR
**APT**	99.5 (87.0–115.2)	8.9 (8.1–10.3)	4.6 (3.7–5.7)	10.2 (8.0–14.0)	0.22 (0.19–0.26)	4.3 (3.2–5.1)
**GLAD**	179.5 (139.5–210.0)	7.1 (6.6–8.5)	6.2 (4.4–8.3)	6.0 (5.0–8.0)	0.20 (0.17–0.26)	2.8 (2.3–3.4)
**PennCNV**	75.0 (63.2–84.5)	21.7 (17.3–25.8)	5.1 (4.0–6.5)	25.0 (23.0–29.2)	0.18 (0.14–0.21)	5.5 (4.8–6.6)
**QuantiSNP**	160.5 (137.8–184.5)	9.0 (8.3–10.0)	8.2 (5.7–23.2)	6.0 (5.9–7.0)	0.23 (0.20–0.26)	3.1 (2.6–3.6)
**R-gada**	211.0 (177.8–236.5)	8.0 (7.1–9.6)	121.0 (18.9–281.4)	7.0 (6.8–10.0)	0.28 (0.23–0.33)	4.4 (3.6–5.4)
**VEGA**	158.5 (137.8–185.2)	7.0 (6.3–7.6)	6.2 (4.7–7.9)	7.0 (5.0–8.0)	0.28 (0.23–0.31)	3.6 (3.1–4.6)
**Algorithm Type**						
**HMM**	98.0 (87.0–111.5)	9.7 (8.9–11.2)	5.2 (4.1–6.6)	10.8 (8.0–14.0)	0.21 (0.19–0.24)	4.3 (3.3–5.0)
**Segmentation**	182.0 (150.8–210.0)	7.4 (6.6–8.1)	7.0 (5.0–8.7)	7.0 (6.0–8.0)	0.26 (0.22–0.30)	3.6 (3.1–4.4)

Given are the median and, in parentheses, the inter-quartile range per sample. **DDR:** Ratio of deletions to duplications.

**Fig 2:**
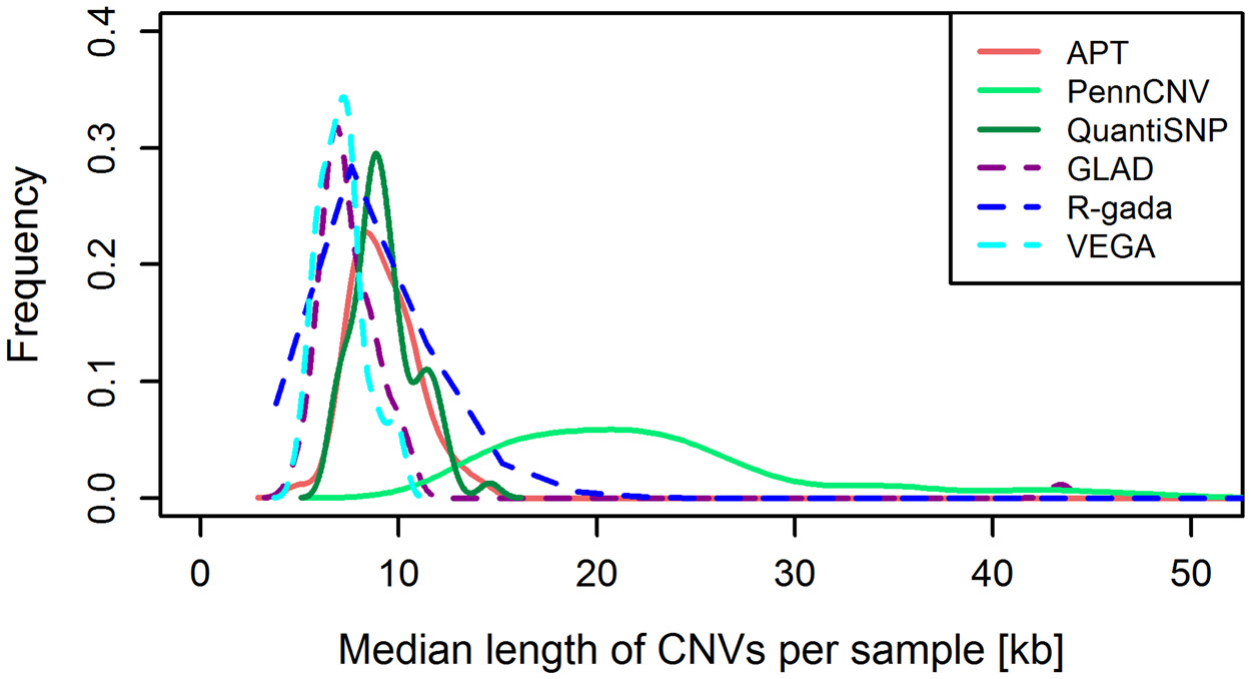
Median sample-specific CNV length. Kernel-smoothed histogram of the median CNV length per sample. Outliers were excluded. **Solid line:** HMM-based algorithms; **dotted lines:** segmentation algorithms.

The distribution of the median CNV length per sample was found to be skewed for all six tools, including some outlier samples with exceptionally long CNVs (Fig 2). In particular, R-gada yielded median CNV lengths of up to 1.9 Mb per sample and predicted CNVs comprising up to 126 Mb. The median of the sample-wise median lengths, taken over all CNVs predicted, was found to be similar for all tools except PennCNV, which showed a trend towards longer CNVs. In general, HMM-based tools tended to yield longer CNVs per sample (median length: 9.7 kb) than segmentation algorithms (7.4 kb, p = 1.6×10–11; Table 1). The cumulative CNV length per sample also differed greatly between tools, ranging from a median of 4.6 Mb (IQR: 3.7–5.7) for APT via 8.1 Mb (5.7–23.2) for QuantiSNP to 121.0 Mb (18.9–281.4) for R-gada. The median cumulative CNV length per sample was consistently larger for Europeans than for Africans (p<0.05 for all tools; S1 Table).

The median number of markers included in a CNV was similar for the different software tools except for PennCNV which, on average, included three times as many markers in a CNV as the other tools. Consequently, PennCNV also exhibited the smallest median inter-marker distance per sample (Table 1). Notably, all six tools were characterized by a median inter-marker distance within CNVs that was well below the overall median of the Affymetrix Human SNP Array 6.0 (684 bp), which is consistent with a preferential prediction of CNVs in regions of increased marker density. Inter-marker distance within CNVs did not differ significantly between Europeans and Africans (S1 Table).

All six tools predicted many more deletions than duplications. The median deletions-to-duplications ratio (DDR) per sample ranged from 2.8 for GLAD to 5.5 for PennCNV (Table 1). HMM-based tools yielded higher DDR values than segmentation algorithms (4.3 vs. 3.6, p = 6.9×10–4; Table 1). No consistent differences in DDR value were noted between European and African samples (S1 Table).

### In-silico validation of predicted CNVs using family information

In view of the observed discrepancies in CNV prediction between different tools, we sought to validate in silico the CNVs predicted for the children using the raw signal intensity data available for the parents. More specifically, we predicted CNVs in the two parents and regarded an offspring CNV as validated if it overlapped by least 90% with a parental CNV of the same state (i.e. gain or loss), predicted by the same tool.

The proportion of CNVs that could be validated per sample differed greatly between tools, with a median percentage over samples that ranged from 41.1% with R-gada to 60.9% with PennCNV (Table 2). HMM-based algorithms yielded more validated CNVs than segmentation algorithms, both in general (55.9% vs. 41.4%) and at the level of the individual tool (Table 2). This trend was apparent for deletions and duplications alike (Table 2). Slightly more deletions than duplications were validated in the case of PennCNV, QuantiSNP and R-gada (median DDR>1) whereas slightly more duplications were validated for APT, GLAD and VEGA (median DDR<1). Nevertheless, the inter-quartile range of the DDR value among validated CNVs included unity for all six tools. The percentage of validation was largely independent of CNV size, evidenced by highly similar validation rates across different bins of CNV size (S1 Fig). In order to assess the impact of false-negative validations that are due to false-negative CNV predictions in the parents, we additionally considered a CNV in the offspring to be validated if at least one out of the six tools predicted a CNV in a parent with an overlap of at least 90% (“extended validation”). Not surprisingly, total numbers and validation rates increased throughout by ~10–20%, but the difference in validation rates between the software tools remained largely unchanged (S4 Table). Features of CNVs with extended validation were very similar to those validated by only a single tool (S5 Table).

**Table 2:** Sample-specific CNV validation rate.

Software	Validated CNVs	Validated deletions [%]	Validated duplications [%]	DDR, confined to validated CNVs	Validated cumulative sequence [%]
**APT**	56.3 (50.8–61.3)	55.7 (50.9–61.1)	60.0 (48.0–67.2)	0.9 (0.8–1.2)	55.7 (42.2–66.9)
**GLAD**	46.0 (39.1–53.2)	46.3 (35.9–52.5)	54.8 (41.0–60.9)	1.3 (1.0–1.5)	45.1 (31.7–58.2)
**PennCNV**	60.9 (53.9–69.5)	64.4 (57.4–74.1)	52.2 (44.3–59.0)	1.2 (0.9–1.4)	56.0 (43.4–65.1)
**QuantiSNP**	50.5 (46.3–56.6)	52.2 (47.2–58.2)	46.4 (37.9–55.7)	1.0 (0.8–1.2)	53.8 (32.3–77.5)
**R-gada**	34.8 (20.4–41.6)	34.6 (20.8–43.1)	32.3 (22.3–44.0)	0.8 (0.6–1.0)	5.2 (1.4–16.4)
**VEGA**	41.1 (33.6–45.9)	39.2 (31.1–45.4)	47.3 (36.6–54.9)	0.9 (0.7–1.1)	36.0 (21.2–53.0)
**Algorithm Type**					
**HMM**	55.9 (49.5–61.3)	57.5 (50.6–60.6)	51.9 (46.1–60.0)	1.1 (0.9–1.3)	55.3 (43.4–65.0)
**Segmentation**	41.4 (35.1–47.6)	40.5 (33.9–46.1)	45.0 (36.0–53.5)	0.9 (0.7–1.1)	34.9 (19.9–47.2)

Given are the median and, in parentheses, the inter-quartile range per sample. **DDR:** Ratio of deletions to duplications.

We also sought for external technical verification from the Database of Genomic Variants (DGV). Sequencing-based CNV data was available for four CEU samples (NA07048, NA10847, NA108 51, NA12878) and two YRI samples (NA19129, NA19240). Unfortunately, complete data were not available for any of the 60 trios studies here. Sequencing-based verification yielded substantially low validation rates between 26% for VEGA to 36% for PennCNV (S2 Fig).

No population differences in validation efficacy were observed (S2 Table). For HMM-based tools, a median of 55.3% of the total genomic sequence included in offspring CNVs was also included in at least one parental CNV. Segmentation-based methods performed substantially worse in this respect. Their median proportion of validated CNV sequence per offspring sample was as low as 5.2% for R-gada (Table 2). However, this abnormality was due to a number of very large CNVs predicted by R-gada that could not be validated.

### Features of validated CNVs

**Fig 3:**
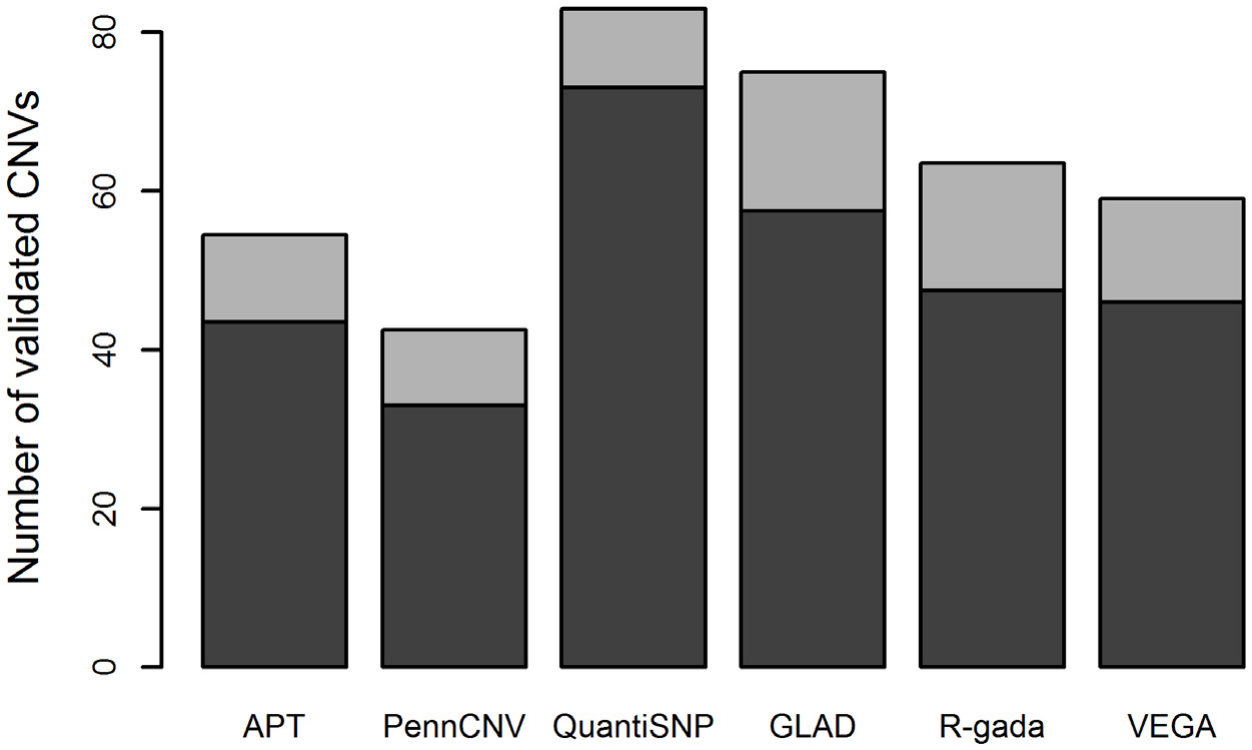
Median sample-specific number of validated CNVs. **Light grey:** duplications; **dark grey:** deletions.

Validated CNVs differed from non-validated CNVs with respect to their total number, their median length, the median number of markers included in a CNV, and the average inter-marker distance. Validated CNVs tended to be longer and more densely covered with markers than non-validated CNVs (Table 3, S3 Table). The median number of validated CNVs per sample ranged from 42.5 (PennCNV) to 83.5 (QuantiSNP). Observed DDR values per sample were similar for validated and non-validated CNVs, with a median for validated CNVs ranging from 3.0 for QuantiSNP and VEGA to 6.2 for PennCNV (Table 3, S3 Table, Fig 3).

**Table 3:** Sample-specific features of validated CNVs.

Software	Total number	Median length [kb]	Median cumulated length [Mb]	Median number of Markers in CNV	Median inter-marker distance [kb]	DDR
**APT**	55.0 (48.8–60.0)	10.3 (9.1–12.0)	2.3 (1.9–3.1)	16.0 (14.0–18.0)	0.19 (0.15–0.21)	3.7 (3.1–4.6)
**GLAD**	79.0 (62.0–94.2)	8.7 (7.3–9.5)	2.7 (1.8–3.5)	10.0 (9.0–12.0)	0.15 (0.11–0.16)	3.6 (2.8–4.1)
**PennCNV**	42.5 (37.8–51.0)	21.2 (16.1–26.0)	2.4 (2.0–3.3)	26.5 (23.9–31.1)	0.15 (0.12–0.19)	6.2 (5.3–8.9)
**QuantiSNP**	83.5 (69.5–95.0)	9.7 (8.9–11.0)	3.2 (2.2–11.0)	9.0 (7.5–9.6)	0.19 (0.13–0.21)	3.0 (2.5–3.7)
**R-gada**	66.0 (37.0–87.0)	8.9 (7.7–10.3)	3.4 (1.4–5.1)	12.0 (10.0–15.2)	0.14 (0.12–0.19)	3.6 (2.9–4.2)
**VEGA**	62.0 (48.5–74.0)	8.2 (7.2–9.2)	2.3 (1.4–3.6)	11.0 (9.0–14.5)	0.16 (0.12–0.20)	3.0 (2.2–4.1)
**Algorithm Type**						
**HMM**	56.0 (49.5–61.0)	10.7 (9.8–12.2)	2.4 (2.0–3.2)	16.0 (14.4–18.0)	0.16 (0.13–0.20)	3.9 (3.3–5.2)
**Segmentation**	70.5 (57.0–79.2)	8.6 (7.6–9.3)	2.5 (1.6–3.5)	11.0 (9.8–13.0)	0.14 (0.12–0.18)	3.2 (2.9–3.8)

Given are the median and, in parentheses, the inter-quartile range per sample. **DDR:** Ratio of deletions to duplications,

### CNV validation by chance alone?

In some cases, family-based validation of a CNV may have occurred by chance alone, and may not have been due to the inheritance of the respective CNV by the offspring. To assess the likelihood of such “pseudo-validation”, we repeatedly permutated the assignment of parents to offspring and analyzed the resulting trios as described above. We performed ten replications, each time evaluating the median proportion of validated CNVs. Unexpectedly, a substantial proportion of CNVs was indeed found to be pseudo-validated in these analyses with a median, over replicates, of the median proportion per sample that ranged from 13.6% (R-gada) to 20.3% (APT) (Table 4). Pseudo-validation rates were higher when parents were permutated within the original population than across. The software-specific median validation proportion ranged from 16.8% to 29.2% in Europeans and from 14.3% to 24.1% in Africans (Table 4).

**Table 4:** Family-based CNV validation by chance alone?

Software	CEU + YRI combined [%]	CEU only [%]	YRI only [%]
**APT**	20.3 (20.1–20.7)	29.2 (29.0–30.6)	24.1 (23.9–24.5)
**GLAD**	16.5 (15.4–16.8)	24.2 (23.9–24.6)	19.9 (18.8–19.9)
**PennCNV**	18.6 (18.3–18.9)	23.5 (23.1–24.0)	16.3 (16.0–17.2)
**QuantiSNP**	16.8 (16.4–17.1)	18.5 (18.1–18.9)	15.7 (14.9–16.2)
**R-gada**	13.6 (13.0–13.9)	16.8 (16.4–18.0)	16.6 (15.9–17.0)
**VEGA**	13.7 (13.1–13.9)	22.5 (22.3–23.2)	14.3 (14.2–14.9)
**Algorithm Type**			
**HMM**	18.1 (17.7–18.4)	22.9 (22.7–24.0)	17.6 (16.9–18.2)
**Segmentation**	14.8 (14.2–15.5)	21.8 (21.4–22.9)	16.5 (15.8–16.9)

Given are the median and, in parentheses, the inter-quartile range.

### Pairwise concordance between tools

A popular albeit heuristic approach to increase the specificity of prediction tools is to use different tools simultaneously. Entities predicted by two different algorithms are usually thought to deserve higher confidence than entities predicted by one algorithm only. In order to assess the concordance between pairs of CNV detection tools, we determined, for each of the 60 offspring individuals from the HapMap trios, the proportion of genomic sequence included in validated CNVs predicted by one tool (‘predictor’) that was also included in validated CNVs predicted by another tool (‘verifier’). Note that this definition of concordance is not necessarily symmetric. Comparisons employed the median proportion (i) of concordant sequence per CNV per sample and (ii) of cumulated concordant CNV sequence per sample. A CNV was considered verified by another algorithm if the proportion of concordant sequence exceeded 90%.

The level of concordance differed greatly between pairs of tools (S7 Table). Thus, the largest median proportion of concordant sequence per CNV per sample was observed for PennCNV as predictor and QuantiSNP as verifier (73.2%). Notably, QuantiSNP as predictor yielded a much lower level of concordance with PennCNV as verifier (40.1%). In general, GLAD as a predictor yielded the highest level of verification by any other tool (52.9–63.7%) whereas QuantiSNP was least verified (40.0–41.4%). The proportion of verified CNVs per sample showed a similar trend and ranged from 32.5% (R-gada as predictor, PennCNV as verifier) to 68.3% (PennCNV, QuantiSNP) and (S6 Table). Use of verification thresholds other than 90% yielded similar results. The median of the proportion of verified cumulated CNV sequence ranged from 14.2% (QuantiSNP as predictor, R-gada as verifier) to 67.76% (VEGA checked by R-gada), but no general trend towards a single tool showing a consistently high level of verification by the other tools was apparent (S7 Table).

## Discussion

Copy-number variation (CNV) has been implicated in the etiology of many complex diseases. While CNV detection is increasingly being based upon next-generation sequencing (NGS) data (see Zhao et al., 2013 [59], for a comprehensive review), NGS-based CNV detection is still faced with a number of issues, including the substantially lower data quality compared to SNP genotyping arrays and problems in detecting forms of structural variation other than deletions, likely contributing to the lack of benchmarking studies for NGS-based CNV detection. On the other hand, many studies still infer CNVs from genome-wide SNP array data. As a legacy of the era of genome-wide association studies, data from this platform is readily available for large sample collections but still not analyzed to its full potential. One reason might be the lack of comparative studies of CNV prediction software. In order to assess the reliability of such CNV detection, we evaluated six frequently used software tools drawing upon parental information for CNV validation. In addition, we investigated the potential for population differences in CNV prediction. Finally, we assessed a common albeit heuristic approach to increase the specificity of CNV detection, namely reliance upon concordant predictions. One important limitation of family-based validation is the inability to detect unusual inheritance patterns of multi-allelic CNVs. Although these are believed to be rare, it is difficult to assess their impact on this study.

The six software tools studied showed major differences in terms of the number and length of CNVs predicted. This discrepancy should raise serious concerns about the general validity of the respective results. Indeed, our family-based validation study revealed a trend for Hidden-Markov models (HMM) to predict fewer CNVs of consistently higher validity than segmentation-based software. In fact, HMM-based software PennCNV predicted the smallest number of CNVs, but achieved the highest level of validation of all tools considered. The six programs also differed in terms of the number and features of the validated CNVs, although these differences were similar to those seen for non-validated CNVs. The median number of CNVs per individual predicted by PennCNV in this study was nearly four times higher than the number reported by Wang et al. [28]. Similarly, we observed a longer CNV length in the predictions of PennCNV. When comparing these results, it has to be considered, that different samples and, more importantly, different array technologies were used. The Illumina HumanHap550 BeadChip [60] used by Wang et al. uses half a million marker (median distance 3 kb), whereas the Affymetrix Human SNP Array 6.0 [44] uses two million marker (median distance 684 bp). This alone is likely lead to major differences in the software performance. Our study thus confirms previous reports of a generally low validity and high false-positive rate of array-based CNV detection, and a preferential prediction of deletions over duplications [17–20]. While the presented validation rates may serve as a proxy for specificity, the use of real-world data with unknown underlying CNVs structure in our study prevents an assessment of sensitivity. In order to compensate for this limitation, we compared the software-specific predictions against a consensus call set consisting of all regions that were predicted to be CNVs by at least three different tools. Sequencing-based verification of six samples using DGV data yielded rather low rates similar to those reported by Pinto et al., 2011 [21]. The observed differences in length of the CNV predictions between algorithms classes are consistent with the observation that segmentation-based algorithms tend to fragment larger CNVs into smaller predictions. This, however, is unlikely to affect the cross comparison of software tools (see below), given that only the amount of covered sequence is crucial for verifying a CNV, not the continuity between segments.

The population origin of a sample played only a minor role in CNV prediction. Anyhow, all software tools except PennCNV showed a trend towards the prediction of fewer and longer CNVs in Europeans than in Africans. This finding may be explicable by a higher level of overall genetic heterogeneity among Africans than non-Africans. Notably, however, the rate of CNV validation was virtually the same in both populations. Somewhat unexpectedly, the likelihood of a chance CNV validation was found to be high and was even increased when European and African trios were considered separately in the assignment of ‘random’ parents. This observation may point towards a population-specific distribution of CNVs, consistent with previous reports [1,32–34]. However, it should be noted that the efficacy of CNV detection hinges on the distribution of the markers used for prediction, which is likely to be population-specific by itself.

The pairwise concordance between tools was often high, but not necessarily symmetric. In particular, PennCNV was superior to all other tools with regard to the median proportion of both the number and concordant sequence of verified CNVs. This renders PennCNV the first choice for initial CNV prediction if specificity is most important. On the other hand, QuantiSNP had the second highest validation rate but showed low concordance with other tools, suggesting that PennCNV and QuantiSNP could be used jointly in order to detect different sets of CNVs.

A high false-positive rate, high probability of chance validation and an insufficient level of concordance CNV prediction between different algorithms as observed in our study would have two important implications for CNV detection. First, CNVs require independent experimental validation, even if predicted concordantly by different algorithms, as has been suggested before by Winchester et al. (2009) [18]. Second, the marker distribution appears to be critical for the ability to predict CNVs reliably. For example, any determination of the breakpoints of a CNV may be difficult in genomic regions that are poorly covered by markers.

The above shortcomings notwithstanding, the high validation rate attained by HMM-based software still render the respective tools a promising means of CNV detection if followed by validation by another method. We thus recommend use of HMM–based tools such as PennCNV and QuantiSNP, perhaps in combination, to achieve high specificity. Anyhow, in view of the large collections of SNP array data that are available from past genome-wide association studies and the still numerous issues with NGS-based CNV detection, a systematic reanalysis of these data aiming at CNV detection seems a worthwhile effort.

## Supporting Information

S1 Fig Rate of validation stratified by the size of the validated CNV. See main text for details.
(TIFF)

S2 Fig Rate of validation using a sequencing data set from the Database of Genomic Variants (DGV). See main text for details.
(TIFF)

S1 File Software commands used for CNV prediction. (PDF)

S1 Table Sample-specific features of predicted CNVs in Africans and Europeans, respectively. (PDF)

S2 Table Sample-specific CNV validation rates in Africans and Europeans, respectively. (PDF)

S3 Table Sample-specific features of non-validated CNVs. (PDF)

S4 Table Sample-specific rate of extended parental CNV validation (i.e. by any of the six software tools). (PDF)

S5 Table Sample-specific features of CNV validated in parents by any of the six software tools (“extended validation”). (PDF)

S6 Table Percentage of verified CNVs per individual in cross-software comparison. (PDF)

S7 Table Concordance of CNV prediction in cross-software comparison. (PDF)

## References

[1] Redon R, Ishikawa S, Fitch KR, Feuk L, Perry GH, Andrews TD, et al Global variation in copy number in the human genome. Nature. 2006;444: 444–54. 10.1038/nature05329 17122850 PMC2669898

[2] Conrad DF, Pinto D, Redon R, Feuk L, Gokcumen O, Zhang Y, et al Origins and functional impact of copy number variation in the human genome. Nature. Nature Publishing Group; 2010;464: 704–12. 10.1038/nature08516 PMC3330748 19812545

[3] Maher B. Personal genomes: The case of the missing heritability. Nature. 2008;456: 18–21. 10.1038/456018a 18987709

[4] Manolio TA, Collins FS, Cox NJ, Goldstein DB, Hindorff LA, Hunter DJ, et al Finding the missing heritability of complex diseases. Nature. 2009;461: 747–53. 10.1038/nature08494 19812666 PMC2831613

[5] Visscher PM, Brown M a, McCarthy MI, Yang J. Five years of GWAS discovery. Am J Hum Genet. The American Society of Human Genetics; 2012;90: 7–24. 10.1016/j.ajhg.2011.11.029 PMC3257326 22243964

[6] McCarroll SA, Huett A, Kuballa P, Chilewski SD, Landry A, Goyette P, et al Deletion polymorphism upstream of IRGM associated with altered IRGM expression and Crohn’s disease. Nat Genet. 2008;40: 1107–12. 10.1038/ng.215 19165925 PMC2731799

[7] The Wellcome Trust Case Control Consortium. Genome-wide association study of CNVs in 16,000 cases of eight common diseases and 3,000 shared controls. Nature. Nature Publishing Group; 2010;464: 713–20. 10.1038/nature08979 PMC2892339 20360734

[8] De Cid R, Riveira-Munoz E, Zeeuwen PLJM, Robarge J, Liao W, Dannhauser EN, et al Deletion of the late cornified envelope LCE3B and LCE3C genes as a susceptibility factor for psoriasis. Nat Genet. 2009;41: 211–5. 10.1038/ng.313 19169253 PMC3128734

[9] Coe BP, Girirajan S, Eichler EE. The genetic variability and commonality of neurodevelopmental disease. Am J Hum Genet. 2012;160C: 118–29. 10.1002/ajmg.c.31327 PMC4114147 22499536

[10] Bochukova EG, Huang N, Keogh J, Henning E, Purmann C, Blaszczyk K, et al Large, rare chromosomal deletions associated with severe early-onset obesity. Nature. Nature Publishing Group; 2010;463: 666–70. 10.1038/nature08689 PMC3108883 19966786

[11] Kathiresan S, Voight BF, Purcell S, Musunuru K, Ardissino D, Mannucci PM, et al Genome-wide association of early-onset myocardial infarction with single nucleotide polymorphisms and copy number variants. Nat Genet. 2009;41: 334–41. 10.1038/ng.327 19198609 PMC2681011

[12] Stefansson H, Rujescu D, Cichon S, Pietiläinen OPH, Ingason A, Steinberg S, et al Large recurrent microdeletions associated with schizophrenia. Nature. 2008;455: 232–6. 10.1038/nature07229 18668039 PMC2687075

[13] Sebat J, Lakshmi B, Malhotra D, Troge J, Lese-Martin C, Walsh T, et al Strong association of de novo copy number mutations with autism. Science (80-). 2007;316: 445–9. 10.1126/science.1138659 PMC2993504 17363630

[14] Carter NP. Methods and strategies for analyzing copy number variation using DNA microarrays. Nat Genet. 2007;39: S16–21. 10.1038/ng2028 17597776 PMC2697494

[15] Mills RE, Walter K, Stewart C, Handsaker RE, Chen K, Alkan C, et al Mapping copy number variation by population-scale genome sequencing. Nature. 2011;470: 59–65. 10.1038/nature09708 21293372 PMC3077050

[16] Coin LJM, Cao D, Ren J, Zuo X, Sun L, Yang S, et al An exome sequencing pipeline for identifying and genotyping common CNVs associated with disease with application to psoriasis Bioinformatics. Oxford, England; 2012;28: i370–i374. 10.1093/bioinformatics/bts379 22962454 PMC3436806

[17] Baross A, Delaney AD, Li HI, Nayar T, Flibotte S, Qian H, et al Assessment of algorithms for high throughput detection of genomic copy number variation in oligonucleotide microarray data. BMC Bioinformatics. 2007;8: 368 10.1186/1471-2105-8-368 17910767 PMC2148068

[18] Winchester L, Yau C, Ragoussis J. Comparing CNV detection methods for SNP arrays. Brief Funct Genomic Proteomic. 2009;8: 353–66. 10.1093/bfgp/elp017 19737800

[19] Zhang D, Qian Y, Akula N, Alliey-Rodriguez N, Tang J, Gershon ES, et al Accuracy of CNV Detection from GWAS Data. PLoS One. 2011;6: e14511 10.1371/journal.pone.0014511 21249187 PMC3020939

[20] Eckel-Passow JE, Atkinson EJ, Maharjan S, Kardia SLR, de Andrade M. Software comparison for evaluating genomic copy number variation for Affymetrix 6.0 SNP array platform. BMC Bioinformatics. BioMed Central Ltd; 2011;12: 220 10.1186/1471-2105-12-220 PMC3146450 21627824

[21] Pinto D, Darvishi K, Shi X, Rajan D, Rigler D, Fitzgerald T, et al Comprehensive assessment of array-based platforms and calling algorithms for detection of copy number variants. Nat Biotechnol. 2011;29: 512–20. 10.1038/nbt.1852 21552272 PMC3270583

[22] Nannya Y, Sanada M, Nakazaki K, Hosoya N, Wang L, Hangaishi A, et al A robust algorithm for copy number detection using high-density oligonucleotide single nucleotide polymorphism genotyping arrays. Cancer Res. 2005;65: 6071–9. 10.1158/0008-5472.CAN-05-0465 16024607

[23] Zhao X, Li C, Paez JG, Chin K, Jänne PA, Chen T, et al An integrated view of copy number and allelic alterations in the cancer genome using single nucleotide polymorphism arrays. Cancer Res. 2004;64: 3060–71. Available: http://www.ncbi.nlm.nih.gov/pubmed/15126342. 15126342 10.1158/0008-5472.can-03-3308

[24] Hupé P, Stransky N, Thiery J-P, Radvanyi F, Barillot E. Analysis of array CGH data: from signal ratio to gain and loss of DNA regions. Bioinformatics. 2004;20: 3413–22. 10.1093/bioinformatics/bth418 15381628

[25] Kidd JM, Cooper GM, Donahue WF, Hayden HS, Sampas N, Graves T, et al Mapping and sequencing of structural variation from eight human genomes. Nature. 2008;453: 56–64. 10.1038/nature06862 18451855 PMC2424287

[26] Korbel JO, Urban AE, Affourtit JP, Godwin B, Grubert F, Simons JF, et al Paired-end mapping reveals extensive structural variation in the human genome. Science (80-). 2007;318: 420–6. 10.1126/science.1149504 17901297 PMC2674581

[27] Korn JM, Kuruvilla FG, McCarroll S a, Wysoker A, Nemesh J, Cawley S, et al Integrated genotype calling and association analysis of SNPs, common copy number polymorphisms and rare CNVs. Nat Genet. 2008;40: 1253–60. 10.1038/ng.237 18776909 PMC2756534

[28] Wang K, Li M, Hadley D, Liu R, Glessner J, Grant SF a, et al PennCNV: an integrated hidden Markov model designed for high-resolution copy number variation detection in whole-genome SNP genotyping data. Genome Res. 2007;17: 1665–74. 10.1101/gr.6861907 17921354 PMC2045149

[29] Affymetrix Inc. Affymetrix Power Tools Manual (1.14.2) [Internet]. 2012 [cited 6 Jun 2012]. Available: http://media.affymetrix.com/support/developer/powertools/changelog/index.html.

[30] Bengtsson H, Irizarry R, Carvalho B, Speed TP. Estimation and assessment of raw copy numbers at the single locus level. Bioinformatics. 2008;24: 759–67. 10.1093/bioinformatics/btn016 18204055

[31] Scharpf RB, Ruczinski I, Carvalho B, Doan B, Chakravarti A, Irizarry R a. A multilevel model to address batch effects in copy number estimation using SNP arrays. Biostatistics. 2011;12: 33–50. 10.1093/biostatistics/kxq043 20625178 PMC3006124

[32] Li J, Yang T, Wang L, Yan H, Zhang Y, Guo Y, et al Whole genome distribution and ethnic differentiation of copy number variation in Caucasian and Asian populations. PLoS One. 2009;4: e7958 10.1371/journal.pone.0007958 19956714 PMC2776354

[33] Kato M, Kawaguchi T, Ishikawa S, Umeda T, Nakamichi R, Shapero MH, et al Population-genetic nature of copy number variations in the human genome. Hum Mol Genet. 2010;19: 761–73. 10.1093/hmg/ddp541 19966329 PMC2816609

[34] Chen W, Hayward C, Wright AF, Hicks A a, Vitart V, Knott S, et al Copy number variation across European populations. PLoS One. 2011;6: e23087 10.1371/journal.pone.0023087 21829696 PMC3150386

[35] Colella S, Yau C, Taylor JM, Mirza G, Butler H, Clouston P, et al QuantiSNP: an Objective Bayes Hidden-Markov Model to detect and accurately map copy number variation using SNP genotyping data. Nucleic Acids Res. 2007;35: 2013–25. 10.1093/nar/gkm076 17341461 PMC1874617

[36] Pique-Regi R, Cáceres A, González JR. R-Gada: a fast and flexible pipeline for copy number analysis in association studies. BMC Bioinformatics. 2010;11: 380 10.1186/1471-2105-11-380 20637081 PMC2915992

[37] Morganella S, Cerulo L, Viglietto G, Ceccarelli M. VEGA: variational segmentation for copy number detection. Bioinformatics. 2010;26: 3020–7. 10.1093/bioinformatics/btq586 20959380

[38] The International HapMap Consortium. The International HapMap Project. Nature. 2003;426: 789–96. 10.1038/nature02168 14685227

[39] The International HapMap Consortium. A haplotype map of the human genome. Nature. 2005;437: 1299–320. 10.1038/nature04226 16255080 PMC1880871

[40] Thorisson GA, Smith AV, Krishnan L, Stein LD. The International HapMap Project Web site. Genome Res. 2005;15: 1592–1593. 10.1101/gr.4413105 16251469 PMC1310647

[41] The International HapMap Consortium. A second generation human haplotype map of over 3.1 million SNPs. Nature. 2007;449: 851–61. 10.1038/nature06258 17943122 PMC2689609

[42] The International HapMap Consortium. Integrating common and rare genetic variation in diverse human populations. Nature. 2010;467: 52–8. 10.1038/nature09298 20811451 PMC3173859

[43] Affymetrix Inc. Affymetrix Genome-Wide Human SNP Nsp/Sty 6.0 User Guide [Internet]. 2011. Available: http://www.affymetrix.com/.

[44] McCarroll SA, Kuruvilla FG, Korn JM, Cawley S, Nemesh J, Wysoker A, et al Integrated detection and population-genetic analysis of SNPs and copy number variation. Nat Genet. 2008;40: 1166–74. 10.1038/ng.238 18776908

[45] LaFramboise T. Single nucleotide polymorphism arrays: a decade of biological, computational and technological advances. Nucleic Acids Res. 2009;37: 4181–93. 10.1093/nar/gkp552 19570852 PMC2715261

[46] Bengtsson H, Wirapati P, Speed TP. A single-array preprocessing method for estimating full-resolution raw copy numbers from all Affymetrix genotyping arrays including GenomeWideSNP 5 & 6. Bioinformatics. 2009;25: 2149–56. 19535535 10.1093/bioinformatics/btp371 PMC2734319

[47] Peiffer DA, Le JM, Steemers FJ, Chang W, Jenniges T, Garcia F, et al High-resolution genomic profiling of chromosomal aberrations using Infinium whole-genome genotyping. Genome Res. 2006;16: 1136–48. 10.1101/gr.5402306 16899659 PMC1557768

[48] Locke DP, Sharp AJ, McCarroll S a, McGrath SD, Newman TL, Cheng Z, et al Linkage disequilibrium and heritability of copy-number polymorphisms within duplicated regions of the human genome. Am J Hum Genet. 2006;79: 275–290. 10.1086/505653 16826518 PMC1559496

[49] The 1000 Genomes Project Consortium. An integrated map of genetic variation from 1,092 human genomes. Nature. 2012;491: 56–65. 10.1038/nature11632 23128226 PMC3498066

[50] Ahn S-M, Kim T-H, Lee S, Kim D, Ghang H, Kim D-S, et al The first Korean genome sequence and analysis: full genome sequencing for a socio-ethnic group. Genome Res. 2009;19: 1622–9. 10.1101/gr.092197.109 19470904 PMC2752128

[51] Bentley DR, Balasubramanian S, Swerdlow HP, Smith GP, Milton J, Brown CG, et al Accurate whole human genome sequencing using reversible terminator chemistry. Nature. 2008;456: 53–9. 10.1038/nature07517 18987734 PMC2581791

[52] Ju YS, Hong D, Kim S, Park S-S, Kim S, Lee S, et al Reference-unbiased copy number variant analysis using CGH microarrays. Nucleic Acids Res. 2010;38: e190 10.1093/nar/gkq730 20802225 PMC2978381

[53] Kim J-I, Ju YS, Park H, Kim S, Lee S, Yi J-H, et al A highly annotated whole-genome sequence of a Korean individual. Nature. 2009;460: 1011–5. 10.1038/nature08211 19587683 PMC2860965

[54] Levy S, Sutton G, Ng PC, Feuk L, Halpern AL, Walenz BP, et al The diploid genome sequence of an individual human. PLoS Biol. 2007;5: e254 10.1371/journal.pbio.0050254 17803354 PMC1964779

[55] McKernan KJ, Peckham HE, Costa GL, McLaughlin SF, Fu Y, Tsung EF, et al Sequence and structural variation in a human genome uncovered by short-read, massively parallel ligation sequencing using two-base encoding. Genome Res. 2009;19: 1527–41. 10.1101/gr.091868.109 19546169 PMC2752135

[56] Pang AW, MacDonald JR, Pinto D, Wei J, Rafiq MA, Conrad DF, et al Towards a comprehensive structural variation map of an individual human genome. Genome Biol. 2010;11: R52 10.1186/gb-2010-11-5-r52 20482838 PMC2898065

[57] Wang J, Wang W, Li R, Li Y, Tian G, Goodman L, et al The diploid genome sequence of an Asian individual. Nature. 2008;456: 60–5. 10.1038/nature07484 18987735 PMC2716080

[58] Wong L-P, Ong RT-H, Poh W-T, Liu X, Chen P, Li R, et al Deep whole-genome sequencing of 100 southeast Asian Malays. Am J Hum Genet. 2013;92: 52–66. 10.1016/j.ajhg.2012.12.005 23290073 PMC3542459

[59] Zhao M, Wang Q, Wang Q, Jia P, Zhao Z. Computational tools for copy number variation (CNV) detection using next-generation sequencing data: features and perspectives. BMC Bioinformatics. BioMed Central Ltd; 2013;14 Suppl 1: S1. 10.1186/1471-2105-14-S11-S1 PMC3846878 24564169

[60] Gunderson KL, Steemers FJ, Lee G, Mendoza LG, Chee MS. A genome-wide scalable SNP genotyping assay using microarray technology. Nat Genet. 2005;37: 549–554. 10.1038/ng1547 15838508

