# Supplementary material for: Family-Based Benchmarking of Copy Number Variation Detection Software
Source: PLoS One. 2015 Jul 21;10(7):e0133465. doi: 10.1371/journal.pone.0133465 (PMC4510559; doi:10.1371/journal.pone.0133465)
Supplement: S1 File — (PDF) [file pone.0133465.s003.pdf]

S1 File. Software commands used for CNV prediction.

The runtime was calculated for the analysis of 90 CEU samples using a Linux operating system with 1 core and 12 GB of memory. The $ indicates a BASH command prompt and the > an R command prompt.

APT
Runtime: 261 minutes.
Used command:

```
$ apt-copynumber-workflow  \
        --verbose 1 --reference-input GenomeWideSNP_6.hapmap270.na31.r1.a5.ref \
        --cdf-file GenomeWideSNP_6.cdf \
        --chrX-probes GenomeWideSNP_6.chrYprobes \
        --chrY-probes GenomeWideSNP_6.chrXprobes \
        --special-snps GenomeWideSNP_6.specialSNPs \
        --annotation-file GenomeWideSNP_6.na31.annot.db--out-dir $OUTDIR \
        --cnchp-output 'true' \
        --text-output 'true' \
        --cel-files CEL_files  \
        --force 'true'
```

PennCNV
Runtime: 100 minutes.
Used command:

```
$ perl detect_cnv.pl \
        --test \
        --hmmfile "./gw6/lib/affygw6.hmm" \
        --pfbfile custom. hg19.pfb \
        --list "./signalfiles.txt" \
        -out "./penncnv_affy.rawcnv"
```

QuantiSNP
Runtime: 2,564 minutes.
Used command:

```
$ run_quantisnp2.sh ./MATLAB_Compiler_Runtime/v79 \
        --outdir ./output \
        --levels  ./ quantisnp/config/levels-affy.dat \
        --config  ./quantisnp/config/params.dat \
        --sampleid sample \
        --gender gender \
        --input-files  ./file\
        --gcdir  ./b37 \
        --lsetting 2000000 \
        --emiters 10 \
        --plot \
```

```
--genotype \
--isaffy \
--verbose
```

GLAD

Runtime: 198 minutes.

Used command:

```
> individual_signals <- read.table( file=current_file, header=TRUE,  sep="\t", comment.char = "#",
colClasses=c("character","character","numeric","NULL","numeric","NULL","NULL","NULL"))
> names(individual_signals_sub) <- c("MarkerName", "Chromosome", "PosBase", "LogRatio")
> individual_data <- within(individual_signals_sub, {Chromosome <- as.integer(individual_signals_sub$Chromosome)} )
> individual_data <- individual_signals_sub[order(individual_signals_sub$Chromosome, individual_signals_sub$PosBase), ]
> individual_data <- within(individual_signals_sub, {PosOrder <- c(1:dim(individual_signals_sub)[1])} )
```

R-gada

Runtime: 145 minutes.

Used commands:

```
> gada_data <- setupGADAaffy(current_file, NumCols=8, log2ratioCol=5)
> gada_SBL <- SBL(gada_data, aAlpha=0.5, estim.sigma2=TRUE)
> gada_BE <- BackwardElimination(gada_SBL,T=6,MinSegLen=3)
```

VEGA

Runtime: 219 minutes.

Used commands:

```
> vega_input <- within(individual_signals, {ProbeEndPosition <- Position})
> vega_input <- vega_input[, c("Chromosome", "Position", "ProbeEndPosition", "Log2Ratio")]
> names(vega_input) <- c("Chromosome", "Probe Start Position", "Probe End Position", "LRR")
> vega(CNVdata = vega_input, chromosomes = c(1:22, "X", "Y"), out_file_name="output" )
```
