# Supplementary material for: Family-Based Benchmarking of Copy Number Variation Detection Software
Source: PLoS One. 2015 Jul 21;10(7):e0133465. doi: 10.1371/journal.pone.0133465 (PMC4510559; doi:10.1371/journal.pone.0133465)
Supplement: S1 Table — (PDF) [file pone.0133465.s004.pdf]

S1 Table. Sample-specific features of predicted CNVs in Africans and Europeans, respectively.

Software	Total number	Median length [kb]	Median cumulated length [Mb]	Median number of markers in CNV	Median inter-marker distance [kp]	DDR
**CNVs in Africans by software**						
**APT**	111.0 (103.2-120.0)	8.1 (7.6-8.9)	4.3 (3.5-5.4)	8.5 (7.5-9.9)	0.22 (0.20-0.24)	3.8 (2.9-4.4)
**GLAD**	197.0 (148.2-230.2)	6.9 (6.3-7.6)	6.2 (4.4-7.8)	6.0 (5.0-7.0)	0.21 (0.18-0.27)	2.8 (2.4-3.1)
**PennCNV**	73.0 (63.5-79.8)	19.8 (15.9-21.9)	4.1 (3.4-5.3)	25.5 (23.1-27.8)	0.17 (0.14-0.20)	6.6 (5.8-8.3)
**QuantiSNP**	165.5 (134.2-177.8)	8.5 (7.5-9.9)	15.8 (7.1-26.3)	6.0 (5.0-7.0)	0.23 (0.21-0.24)	3.4 (2.9-3.7)
**R-gada**	229.5 (189.0-251.5)	7.4 (6.7-8.4)	73.7 (13.9-139.2)	7.0 (6.0-7.0)	0.26 (0.23-0.29)	4.6 (3.8-5.4)
**VEGA**	185.0 (157.2-195.2)	6.5 (6.1-7.4)	5.5 (4.6-7.6)	5.0 (5.0-7.0)	0.28 (0.26-0.32)	3.8 (3.1-4.6)
**CNVs in Africans by algorithm type**						
**HMM**	108.0 (100.2-119.5)	8.9 (8.5-10.0)	4.6 (3.8-5.4)	8.8 (7.5-10.0)	0.21 (0.19-0.23)	3.8 (3.1-4.4)
**Segmentation**	204.0 (178.2-225.0)	7.0 (6.5-7.6)	7.0 (5.0-8.2)	6.0 (6.0-7.0)	0.26 (0.22-0.30)	3.8 (3.0-4.5)
**CNVs in Europeans by algorithm software**						
**APT**	*87.0 (81.0-94.0)	*10.2 (9.3-10.7)	5.0 (4.1-6.4)	*13.8 (11.1-15.4)	0.22 (0.19-0.31)	*4.9 (3.7-5.8)
**GLAD**	*161.5 (133.8-192.5)	*7.5 (6.8-8.6)	6.1 (4.4-8.6)	*8.0 (6.0-8.9)	0.20 (0.16-0.24)	1.9 (1.5-2.3)
**PennCNV**	67.0 (54.2-76.8)	*24.4 (18.8-33.6)	5.3 (4.3-6.4)	26.0 (25.0-30.0)	0.20 (0.15-0.22)	*4.5 (3.8-5.5)
**QuantiSNP**	*137.0 (122.0-147.5)	*10.0 (8.8-11.2)	*7.7 (4.4-22.4)	6.2 (6.0-7.0)	*0.27 (0.21-0.30)	*2.6 (2.2-3.3)
**R-gada**	*200.0 (174.8-214.0)	*9.3 (7.6-10.4)	*254.7 (45.1-390.1)	*9.0 (7.6-12.5)	*0.31 (0.25-0.39)	*4.1 (3.4-5.4)
**VEGA**	*145.5 (119.0-159.2)	*7.4 (6.9-8.1)	6.5 (4.7-8.0)	*8.0 (6.2-9.0)	0.27 (0.22-0.30)	3.4 (3.1-4.6)
**CNVs in Europeans by algorithm type**						
**HMM**	*87.0 (81.0-94.0)	*10.6 (9.9-12.0)	5.0 (4.3-6.3)	*13.8 (11.1-15.4)	0.22 (0.19-0.27)	4.1 (3.3-5.1)
**Segmentation**	*162.0 (144.0-186.5)	*7.8 (7.1-8.7)	7.1 (5.3-10.3)	*8.0 (7.2-9.4)	0.26 (0.22-0.32)	3.5 (3.1-4.2)

Given are the median and, in parentheses, the inter-quartile range for the offspring of the 30 HapMap YRI trios (Africans) and the 30 HapMap CAU trios (Europeans). Entries denoted by * show a significant difference between Africans and Europeans (Wilcoxon rank sum test p value < 0.05). **DDR:** Ratio of deletions to duplications.
