# Supplementary material for: Family-Based Benchmarking of Copy Number Variation Detection Software
Source: PLoS One. 2015 Jul 21;10(7):e0133465. doi: 10.1371/journal.pone.0133465 (PMC4510559; doi:10.1371/journal.pone.0133465)
Supplement: S2 Table — (PDF) [file pone.0133465.s005.pdf]

S2 Table. Sample-specific CNV validation rates in Africans and Europeans, respectively.

Software	Validated CNVs [%]	Validated deletions [%]	Validated duplications [%]	DDR, confined to validated CNVs	Validated cumulative sequence [%]
**Validation Rates in Africans by software**					
**APT**	53.1 (48.8-59.2)	53.7 (50.6-57.3)	62.6 (48.7-68.2)	0.9 (0.8-1.0)	56.7 (47.1-68.8)
**GLAD**	43.1 (36.5-49.3)	39.9 (34.5-49.5)	53.6 (40.8-58.5)	1.1 (1.0-1.5)	42.5 (28.2-57.1)
**PennCNV**	59.1 (53.0-64.5)	62.5 (54.2-68.9)	52.6 (45.2-60.4)	1.2 (1.0-1.5)	51.9 (40.4-63.6)
**QuantiSNP**	48.9 (45.1-53.5)	50.2 (46.7-53.5)	39.0 (33.3-51.6)	0.9 (0.8-1.0)	66.9 (38.2-86.0)
**R-gada**	37.0 (25.0-44.0)	35.6 (24.8-41.9)	36.7 (24.8-54.4)	0.7 (0.5-0.9)	5.7 (2.1-16.6)
**VEGA**	34.3 (24.6-39.5)	32.5 (22.6-37.1)	42.7 (29.9-51.8)	0.8 (0.6-1.0)	27.0 (15.6-49.6)
**Validation Rates in Africans by algorithm type**					
**HMM**	53.6 (48.8-56.4)	53.6 (48.8-57.7)	50.0 (43.4-58.5)	1.1 (1.0-1.4)	54.5 (48.7-68.7)
**Segmentation**	38.7 (33.5-43.9)	36.0 (31.4-40.7)	42.7 (34.4-53.4)	0.8 (0.7-0.9)	26.9 (15.8-44.6)
**Validation Rates in Europeans by software**					
**APT**	*58.8 (52.2-62.9)	*58.9 (54.8-65.3)	57.1 (47.1-66.7)	1.0 (0.9-1.2)	54.3 (39.5-63.9)
**GLAD**	*50.8 (41.1-56.2)	*49.9 (40.9-57.3)	56.0 (43.3-61.3)	1.1 (1.0-1.3)	51.9 (34.0-58.0)
**PennCNV**	61.1 (51.7-65.8)	64.5 (53.0-72.0)	56.9 (48.6-65.0)	1.1 (0.9-1.3)	48.1 (34.8-61.1)
**QuantiSNP**	48.1 (45.3-53.4)	49.0 (43.8-55.0)	44.1 (38.9-53.4)	*1.1 (1.0-1.4)	41.2 (28.3-79.7)
**R-gada**	29.9 (19.5-40.2)	30.7 (20.1-43.2)	*27.6 (18.9-36.1)	0.9 (0.8-1.2)	4.1 (1.1-16.0)
**VEGA**	*46.0 (42.3-50.6)	*45.6 (41.3-51.1)	49.2 (42.0-55.5)	0.9 (0.7-1.2)	*46.2 (30.2-57.7)
**Validation Rates in Europeans by algorithm type**					
**HMM**	57.1 (50.5-61.7)	57.0 (52.2-62.3)	55.6 (47.9-58.2)	1.1 (0.9-1.2)	50.1 (38.7-61.1)
**Segmentation**	*44.9 (38.9-50.4)	*45.2 (37.6-52.0)	45.5 (39.3-53.5)	1.0 (0.9-1.1)	*44.7 (24.4-51.0)

Given are the median and, in parentheses, the inter-quartile range for the offspring of the 30 HapMap YRI trios (Africans) and the 30 HapMap CAU trios (Europeans). Entries denoted by * show a significant difference between Africans and Europeans (Wilcoxon rank sum test p value < 0.05). **DDR:** Ratio of deletions to duplications.
