# Supplementary material for: Family-Based Benchmarking of Copy Number Variation Detection Software
Source: PLoS One. 2015 Jul 21;10(7):e0133465. doi: 10.1371/journal.pone.0133465 (PMC4510559; doi:10.1371/journal.pone.0133465)
Supplement: S3 Table — (PDF) [file pone.0133465.s006.pdf]

**S3 Table. Sample-specific features of non-validated CNVs.**

| Software | Total number | Median length [kb] | Median cumulated length [Mb] | Median number of markers in CNV | Median inter-marker distance [kp] | DDR |
|---|---|---|---|---|---|---|
| **APT** | 43.0 (34.8 - 57.2) | 8.1 (6.4 - 10.0) | 1.9 (1.4 - 3.3) | 6.2 (4.0 - 10.0) | 0.30 (0.22 - 0.37) | 4.7 (3.3 - 6.5) |
| **GLAD** | 92.0 (63.5 - 127.8) | 6.6 (5.7 - 7.5) | 3.3 (1.9 - 4.9) | 4.0 (4.0 - 5.5) | 0.30 (0.23 - 0.40) | 2.0 (1.2 - 2.9) |
| **PennCNV** | 29.0 (18.0 - 35.2) | 26.2 (17.3 - 44.8) | 2.0 (1.5 - 3.4) | 23.0 (19.4 - 29.0) | 0.25 (0.17 - 0.35) | 4.8 (3.7 - 6.6) |
| **QuantiSNP** | 75.0 (60.0 - 92.2) | 7.9 (6.9 - 10.2) | 3.0 (2.1 - 5.8) | 4.0 (4.0 - 5.5) | 0.31 (0.26 - 0.40) | 2.9 (2.4 - 4.1) |
| **R-gada** | 130.0 (107.5 - 169.2) | 7.6 (6.2 - 8.9) | 100.6 (16.4 - 266.8) | 6.0 (5.0 - 8.1) | 0.35 (0.31 - 0.44) | 5.5 (3.9 - 7.4) |
| **VEGA** | 89.5 (72.8 - 120.2) | 6.0 (5.2 - 7.4) | 3.6 (2.5 - 5.9) | 5.0 (4.0 - 6.0) | 0.37 (0.29 - 0.43) | 4.5 (3.1 - 6.2) |
| **Algorithm Type** | | | | | | |
| **HMM** | 43.0 (34.0 - 52.0) | 9.3 (7.8 - 11.4) | 2.2 (1.7 - 3.6) | 7.0 (5.0 - 10.0) | 0.29 (0.23 - 0.36) | 4.3 (3.2 - 5.2) |
| **Segmentation** | 103.0 (86.2 - 141.5) | 6.7 (5.7 - 7.6) | 4.2 (3.0 - 6.0) | 5.0 (4.0 - 6.0) | 0.35 (0.28 - 0.41) | 4.3 (3.1 - 5.2) |

Given are the median and, in parentheses, the inter-quartile range. **DDR:** Ratio of deletions to duplications.
