# Supplementary material for: Family-Based Benchmarking of Copy Number Variation Detection Software
Source: PLoS One. 2015 Jul 21;10(7):e0133465. doi: 10.1371/journal.pone.0133465 (PMC4510559; doi:10.1371/journal.pone.0133465)
Supplement: S4 Table — (PDF) [file pone.0133465.s007.pdf]

S4 Table. Sample-specific rate of extended parental CNV validation (i.e. by any of the six software tools).

Software	Validated CNVs	Validated deletions [%]	Validated duplications [%]	DDR, confined to validated CNVs	Validated cumulative sequence [%]
**APT**	71.0 (65.3 - 75.4)	71.3 (65.0 - 75.5)	71.4 (61.4 - 83.8)	1.0 (0.8 - 1.2)	73.2 (59.7 - 83.1)
**GLAD**	63.1 (55.8 - 70.2)	61.8 (52.8 - 69.1)	71.4 (65.7 - 78.4)	1.2 (1.0 - 1.4)	67.3 (57.2 - 77.5)
**PennCNV**	76.7 (73.0 - 81.8)	82.0 (76.6 - 85.8)	69.8 (58.9 - 76.6)	1.1 (0.9 - 1.2)	72.5 (57.8 - 79.8)
**QuantiSNP**	63.6 (59.3 - 69.0)	64.9 (58.1 - 69.6)	59.5 (50.0 - 66.7)	1.1 (0.9 - 1.2)	69.6 (42.7 - 83.7)
**R-gada**	45.0 (31.9 - 52.3)	45.5 (31.8 - 52.8)	41.4 (28.4 - 56.7)	0.9 (0.7 - 1.0)	6.5 (1.9 - 23.2)
**VEGA**	54.1 (50.5 - 60.2)	52.5 (46.8 - 59.6)	62.2 (53.7 - 72.1)	0.9 (0.7 - 1.0)	59.9 (42.0 - 71.3)
**Algorithm Type**					
**HMM**	70.5 (67.0 - 75.4)	71.4 (66.1 - 75.5)	68.0 (58.3 - 72.3)	1.1 (0.9 - 1.2)	72.0 (59.7 - 79.8)
**Segmentation**	53.9 (50.9 - 60.4)	52.7 (47.9 - 59.6)	61.8 (55.1 - 70.4)	0.9 (0.8 - 1.0)	57.8 (35.9 - 66.5)

Given are the median and, in parentheses, the inter-quartile range. **DDR:** Ratio of deletions to duplication
