# Supplementary material for: Family-Based Benchmarking of Copy Number Variation Detection Software
Source: PLoS One. 2015 Jul 21;10(7):e0133465. doi: 10.1371/journal.pone.0133465 (PMC4510559; doi:10.1371/journal.pone.0133465)
Supplement: S5 Table — (PDF) [file pone.0133465.s008.pdf]

S5 Table. Sample-specific features of CNV validated in parents by any of the six software tools ("extended validation").

Software	Total number	Median length [kb]	Median cumulated length [Mb]	Median number of markers in CNV	Median inter-marker distance [kp]	DDR
**APT**	70.0 (64.0 - 75.2)	10.0 (9.1 - 11.7)	3.4 (2.6 - 4.0)	15.0 (13.0 - 17.5)	0.19 (0.15 - 0.22)	4.0 (3.1 - 4.8)
**GLAD**	113.0 (88.5 - 127.8)	8.6 (7.3 - 9.5)	4.1 (2.9 - 5.4)	9.0 (8.0 - 11.0)	0.15 (0.13 - 0.18)	3.3 (2.7 - 3.9)
**PennCNV**	57.0 (47.0 - 64.2)	19.8 (16.5 - 24.3)	3.5 (2.7 - 4.4)	26.0 (23.0 - 29.6)	0.16 (0.13 - 0.20)	6.1 (4.9 - 8.0)
**QuantiSNP**	102.0 (86.5 - 115.8)	9.8 (8.9 - 11.1)	5.0 (2.8 - 11.7)	8.0 (7.0 - 10.0)	0.20 (0.15 - 0.21)	3.2 (2.6 - 3.7)
**R-gada**	90.0 (49.0 - 105.8)	9.1 (8.3 - 10.1)	5.0 (2.3 - 6.8)	11.8 (9.5 - 14.6)	0.16 (0.13 - 0.20)	3.7 (3.0 - 4.2)
**VEGA**	87.5 (71.5 - 101.0)	8.2 (7.0 - 9.0)	3.6 (2.4 - 4.5)	10.0 (8.0 - 13.1)	0.19 (0.15 - 0.22)	3.2 (2.5 - 4.1)
**Algorithm Type**						
**HMM**	70.0 (64.8 - 75.0)	10.8 (9.7 - 12.3)	3.5 (2.6 - 4.3)	15.0 (13.8 - 17.5)	0.18 (0.15 - 0.20)	4.0 (3.3 - 4.9)
**Segmentation**	94.0 (76.0 - 104.2)	8.6 (7.7 - 9.4)	3.8 (2.7 - 5.0)	10.0 (9.0 - 12.0)	0.17 (0.14 - 0.20)	3.4 (2.9 - 3.8)

Given are the median and, in parentheses, the inter-quartile range. **DDR:** Ratio of deletions to duplications
