# Supplementary material for: Family-Based Benchmarking of Copy Number Variation Detection Software
Source: PLoS One. 2015 Jul 21;10(7):e0133465. doi: 10.1371/journal.pone.0133465 (PMC4510559; doi:10.1371/journal.pone.0133465)
Supplement: S6 Table — (PDF) [file pone.0133465.s009.pdf]

S6 Table. Percentage of verified CNVs per individual in cross-software comparison.

	Verifier						
**Predictor**	**APT**	**GLAD**	**PennCNV**	**QuantiSNP**	**R-gada**	**VEGA**	**3+**
**Verification threshold: 95 %**							
**APT**	-	45.8	42.9	55.6	44.5	64.8	74.0
**GLAD**	60.8	-	48.9	56.2	55.8	57.4	80.0
**PennCNV**	52.8	41.9	-	66.7	40.3	44.0	71.4
**QuantiSNP**	34.9	30.4	38.1	-	35.5	35.2	52.8
**R-gada**	36.6	38.6	30.1	44.8	-	51.0	56.5
**VEGA**	56.5	42.7	33.3	48.4	61.5	-	67.4
**3+**	74.3	71.9	59.9	75.9	85.7	82.8	-
**Verification threshold: 90 %**							
**APT**	-	49.6	44.3	58.1	45.8	66.4	74.2
**GLAD**	61.3	-	50.3	58.1	56.2	58.7	80.0
**PennCNV**	55.4	47.9	-	68.3	42.5	46.1	72.3
**QuantiSNP**	36.2	33.0	38.9	-	36.2	36.5	52.8
**R-gada**	38.8	41.3	32.5	46.0	-	51.4	56.5
**VEGA**	59.2	45.9	34.1	50.0	62.4	-	67.5
**3+**	77.3	79.3	63.1	78.8	90.4	87.2	-
**Verification threshold: 80 %**							
**APT**	-	54.0	45.9	60.9	47.5	69.9	76.7
**GLAD**	62.6	-	51.4	59.2	57.3	61.3	80.7
**PennCNV**	58.0	54.6	-	70.7	46.7	51.0	75.4
**QuantiSNP**	38.5	36.4	39.0	-	38.9	39.6	55.0
**R-gada**	39.9	43.4	33.7	48.8	-	52.0	57.3
**VEGA**	61.1	49.6	36.4	50.6	62.4	-	69.4
**3+**	78.5	83.2	64.2	81.4	91.8	90	-
**Verification threshold: 50 %**							
**APT**	-	59.9	49.7	62.9	50.7	72.7	78.3
**GLAD**	63.8	-	53.3	61.0	59.2	63.0	82.8
**PennCNV**	62.2	61.1	-	74.7	50.5	55.4	80.0
**QuantiSNP**	41.1	41.0	40.0	-	41.6	42.6	57.4
**R-gada**	40.6	47.3	36.8	50.8	-	52.1	58.3
**VEGA**	62.7	54.4	37.9	53.1	63.0	-	71.0
**3+**	80.3	88.2	66.7	84.3	94.1	92.8	-

Median percentage of verified CNVs per individual. **3+**: Set of sample-specific variants that have been concordantly called by at least three tools.
