# Supplementary material for: Family-Based Benchmarking of Copy Number Variation Detection Software
Source: PLoS One. 2015 Jul 21;10(7):e0133465. doi: 10.1371/journal.pone.0133465 (PMC4510559; doi:10.1371/journal.pone.0133465)
Supplement: S7 Table — (PDF) [file pone.0133465.s010.pdf]

S7 Table. Concordance of CNV prediction in cross-software comparison.

	Verifier							
**Predictor**	**APT**	**GLAD**	**PennCNV**	**QuantiSNP**	**R-gada**	**VEGA**	**HMM**	**Segmentation**
**Median percentage of concordant sequence per CNV**								
**APT**	-	58.3	48.7	62.1	49.6	71.3	54.0	57.9
**GLAD**	63.7	-	52.9	60.6	58.3	62.6	59.1	56.0
**PennCNV**	61.0	58.1	-	73.2	50.2	53.2	66.6	52.6
**QuantiSNP**	40.4	40.0	40.1	-	40.9	41.4	41.0	40.2
**R-gada**	40.4	46.1	35.6	50.5	-	52.1	42.6	48.6
**VEGA**	61.9	52.3	37.4	52.5	63.3	-	50.8	55.5
**HMM**	49.9	51.6	44.7	67.5	46.9	55.9	-	40.2
**Segmentation**	55.8	49.6	42.1	54.5	61.7	57.2	59.1	-
**Median percentage of concordant cumulated CNV sequence**								
**APT**	-	65.8	71.7	46.6	51.4	62.9	57.3	58.8
**GLAD**	66.7	-	60.5	38.1	43.9	55.4	54.9	51.8
**PennCNV**	70.7	58.6	-	50.2	46.5	55.4	58.3	51.3
**QuantiSNP**	31.0	29.1	33.9	-	16.6	23.3	34.2	24.9
**R-gada**	42.8	36.5	34.6	24.7	-	47.2	36.0	39.5
**VEGA**	66.7	64.1	55.7	39.6	70.6	-	53.8	64.8
**HMM**	47.7	50.6	52.0	47.0	41.9	50.0	-	24.9
**Segmentation**	57.2	49.3	49.8	34.7	59.3	45.1	54.9	-

Concordance was defined as an identical CN class prediction (gain, loss, normal) at a given locus. Only those CNV predictions that were validated by the use of parental information were included (validation threshold: 90 % overlapping CNV sequence)
